# Secondary Acute Myeloid Leukemia in a One-Year-Old Girl Diagnosed with JAK2-V617F Mutation Positive Myeloproliferative Neoplasm

**DOI:** 10.1155/2014/473297

**Published:** 2014-03-18

**Authors:** Gary M. Woods, Rajinder P. S. Bajwa, Samir B. Kahwash, Terri Guinipero

**Affiliations:** ^1^Department of Hematology/Oncology/BMT, Nationwide Children's Hospital, 700 Children's Drive, Columbus, OH 43205, USA; ^2^Department of Hematology/Oncology/BMT, Nationwide Children's Hospital, The Ohio State University, 700 Children's Drive, Columbus, OH 43205, USA; ^3^Department of Pathology, Nationwide Children's Hospital, The Ohio State University, 700 Children's Drive, Columbus, OH 43205, USA

## Abstract

Myeloproliferative neoplasms (MPNs) are a group of clonal disorders characterized by hyperproliferation of hematologic cell lines and have been associated with tyrosine kinase JAK2-V617F mutations. Secondary acute myeloid leukemia (sAML) is a known complication of JAK2-V617F+ MPNs and bears a poor prognosis. Although the evolution of a JAK2-V617F+ MPN to a mixed-lineage leukemia has been reported in the pediatric population, no evolutions into sAML have been described. We present a case of a one-year-old girl diagnosed with JAK2-V617F+ MPN with evolution into sAML. Despite initial morphologic remission, she eventually relapsed and succumbed to her disease.

## 1. Introduction

Myeloproliferative neoplasms (MPNs) are a heterogeneous group of disorders manifested by increased hematopoiesis and proliferation of one or more of the hematologic cell lines. A mutation in the tyrosine kinase JAK 2 (JAK2-V617F) is frequently encountered in patients with MPNs [1]. Adults with JAK2-V617F+ MPNs can evolve into acute myeloid leukemia (AML). Evolution into AML from a known MPN is included in the category of secondary AML (sAML). Conversely, the JAK2-V617F mutation is more common in sAML than* de novo* AML [2–6]. Secondary AMLs evolving from JAK2-V617F+ MPNs bear a poor prognosis since first complete remission (CR) is difficult to obtain; even matched sibling donor hematopoietic stem cell transplant (HSCT) recipients have poor outcomes [4, 7–9]. JAK2-V617F mutations have been described in pediatric patients with MPNs, but the evolution into sAML has not [10]. We describe a case of a one-year-old girl with a JAK2-V617F+ MPN that evolved into sAML.

## 2. Case Report

An 1-year old female presented with a two-month history of fever, fussiness, and refusal to bear weight. Significant physical findings included a one centimeter, freely mobile, nodular mass under her right eyebrow, diffuse lymphadenopathy, and absence of organomegaly.

**Figure 1:**
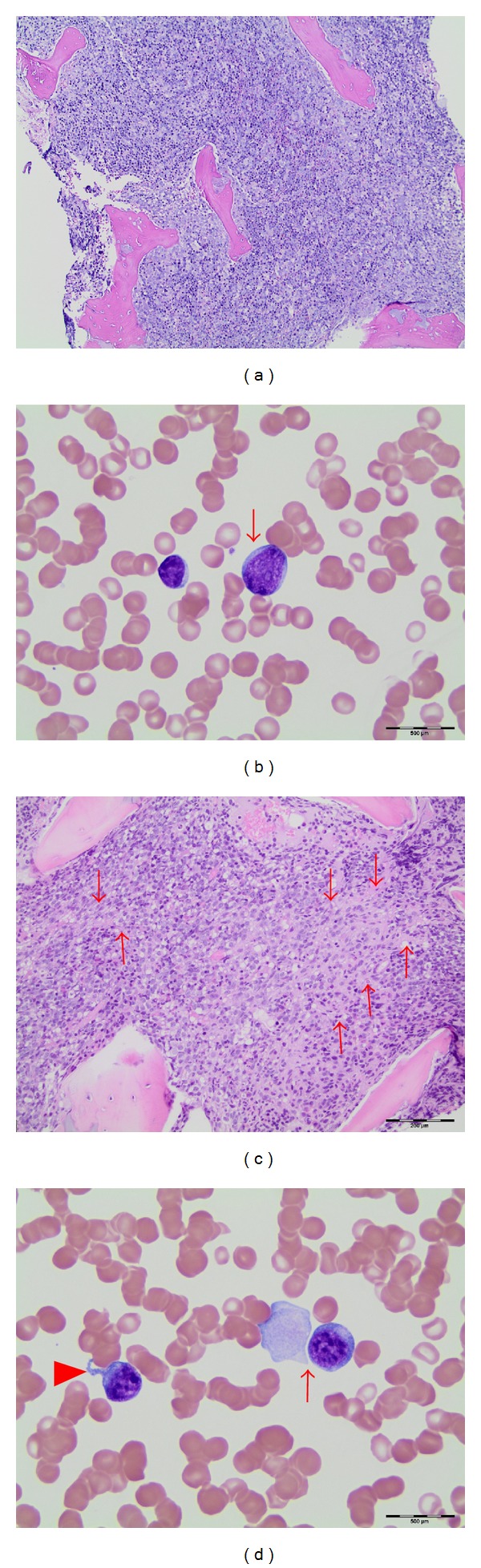
(a) Bone marrow core biopsy showing high cellularity with marrow space occupied mainly by early myeloid cells. (b) Most blasts are nondescript, medium-sized with scanty blue cytoplasm and small nucleoli. (c) Collagen bands (arrows) are evident in areas of core biopsy. (d) Some blasts showed megakaryocytic features with cytoplasmic blebs (arrowhead) or platelet-like pseudopods (arrow).

Initial hematologic evaluation revealed a white blood cell count (WBC) of 16.5 × 10^9^/L, hemoglobin of 9.6 g/dL, and a platelet count of 30 × 10^9^/L. The WBC differential demonstrated a left shift with 2% peripheral blasts. Bone marrow evaluation on hospital day (HD) three was morphologically concerning for myelodysplastic syndrome revealing hypercellularity (Figure 1(a)) with blasts constituting 4% of marrow cells (Figure 1(b)). Cytogenetics revealed 46,XX,dup(8)(q21.3q23), add (11)(p13),del (13)(q12q14),add (18)(p11.2)[5]/46,sl,add(X)(q26),add(3)(p25),add(9)(q32),-del (13), inv (15)(q15sq26.1),+18,-add (18),+mar[11].ish add (11)(MLL+), which, although complex, identified no known specific markers for MPNs, MDS, or AML.

A second bone marrow evaluation was done on HD 11 due to continued pancytopenia and little clinical improvement. At this time, blasts constituted 6% of marrow cells and the morphologic findings met the criteria for the WHO category of refractory anemia with excess blasts. The marrow was also significant for extensive fibrosis (Figure 1(c)) and a few blasts with megakaryocytic features (Figure 1(d)). Cytogenetic findings were similar to the previous study except for chromosomal gains of 7, 21, and X; again, no characteristic AML or MPN abnormalities were identified. Genetic MPN screens, including JAK2-V617F mutation, were initiated secondary to the extensive marrow fibrosis.

**Figure 2:**
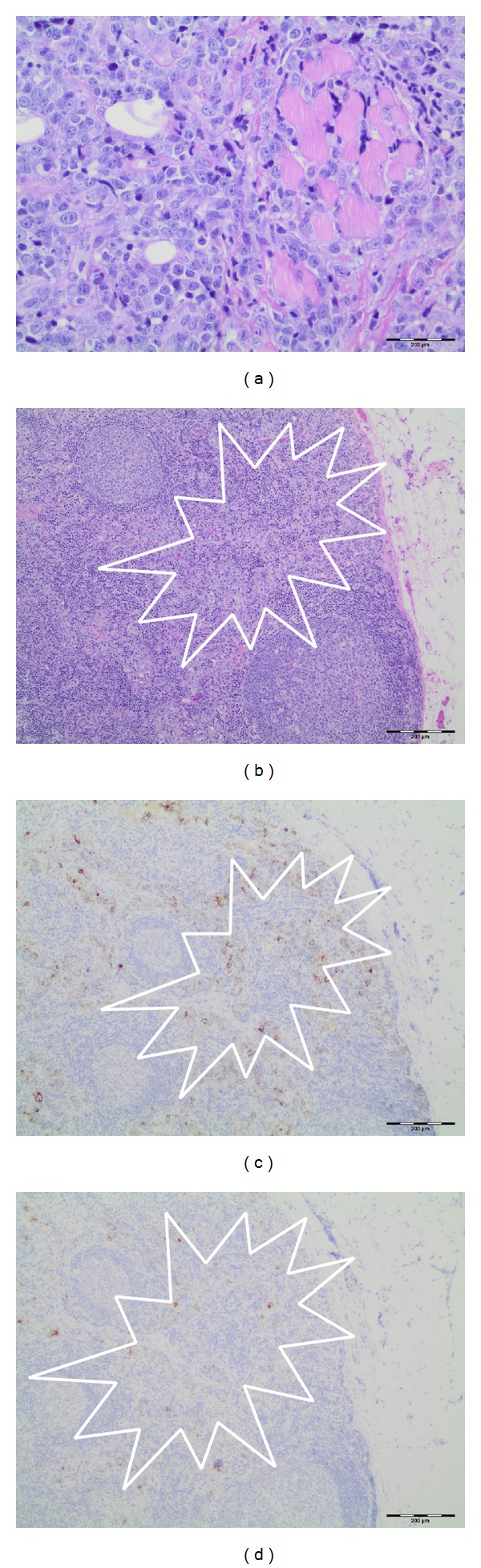
(a) Myeloid sarcoma blasts infiltrating striated muscle in biopsy from eyebrow. (b) Inguinal lymph node showing myeloid blasts in subcapsular and interfollicular sinuses (H&E). (c) Immunoperoxidase staining for CD42b showing positivity in blasts infiltrating lymph node subcapsular and interfollicular sinuses. (d) Immunoperoxidase staining for CD61 showing positivity in blasts infiltrating lymph nose subcapsular and interfollicular sinuses.

As the nodular lesion above her eye continued to enlarge, we elected to biopsy it along with an enlarged inguinal lymph node on HD 13. Both biopsies were consistent with myeloid sarcoma (Figures 2(a), 2(b), 2(c), and 2(d)). A third bone marrow evaluation on HD 19 prior to initiation of chemotherapy was consistent with AML with a myeloid blast percentage of 43%. Immunophenotypic studies by flow cytometry showed blasts positivity for CD61 and CD42b (megakaryocytic markers) along with CD14, CD4, and CD71. Immunoperoxidase staining performed on sections from the core biopsy confirmed specific blasts positivity for CD42b, CD61, and CD117 and the diagnosis of Acute Megakaryocytic Leukemia was rendered. The patient was enrolled on COG protocol AAML1031 and randomized to arm A. MPN genetic studies resulted after completion of induction chemotherapy and revealed JAK2-V617F mutation. Retrospectively, our patient was diagnosed with JAK2-V617F+ MPN with rapid evolution into sAML.

After Induction I, her minimal residual disease (MRD) was 2.8% and JAK2-V617F mutation genetic studies sent on the bone marrow aspirate remained positive. Now considered high risk due to MRD positivity, she continued her chemotherapy per protocol and did obtain morphologic CR after Induction II. She completed Intensification I and per protocol was to proceed to HSCT. A matched unrelated HSCT was planned with a 4/6 human leukocyte antigen allelic matched umbilical cord blood unit. However, while waiting for sufficient count recovery, she developed an upper respiratory tract infection due to parainfluenza virus. We elected to delay HSCT and proceed to Intensification II. Following Intensification II, peripheral blasts were reported and bone marrow examination confirmed relapsed AML with megakaryocytic immunophenotype. Blasts constituted 23% of the marrow cells.

The family opted to continue curative intent therapy and she was given a course of Clofarabine. Peripheral blasts recurred on count recovery and, with refractory disease, the family elected to pursue palliative measures with oral hydroxyurea. She passed away peacefully at home shortly after starting this therapy.

## 3. Discussion

Initially, there was difficulty in obtaining a definitive diagnosis in our patient, as it seems she was actively evolving into sAML at the time of presentation. Her bone marrow aspirate samples revealed blast counts too low for the diagnosis of AML, while changes in the biopsies were consistent with more blast involvement. This was attributed to the extensive marrow fibrosis, raising the possibility of myelofibrosis, which prompted our MPN investigation and JAK2 testing. We only elected to biopsy her eyebrow lesion because her diagnosis was still unclear. Had we proceeded with this biopsy earlier, we would have obtained a myeloid sarcoma diagnosis and begun therapy, and we would not have performed the MPN evaluation. The JAK2-V617F mutation result did not alter her clinical course as she would have been upstaged to high risk due to her MRD status, but it does explain her poor response to therapy. MPN evaluations should be considered when bone marrow examinations are inconclusive in pediatric patients clinically suspicious for leukemia.

The JAK2-V617F mutation resulted after initiating therapy and was not considered a high-risk marker. AML patients with JAK2-V617F mutations are now ineligible for AAML1031, a study for* de novo* AML, since it is associated with MPNs and evolution into sAML [8]. Based on historical evidence, AML patients with JAK2-V617F mutations should be considered sAML and treated accordingly.

High-risk AML patients should receive a HSCT if any donor is available after Intensification I. Upper respiratory parainfluenza infections have been associated with progression to pneumonia and/or pneumonitis in HSCT patients, especially if the infection is obtained prior to engraftment, with reported transplant-related mortality (TRM) rates of up to 70% [11, 12]. Although our patient was in CR, proceeding to HSCT with a known viral infection would have significantly increased her TRM risk; thus, her transplant was delayed until repeat viral testing was negative.

Two children have been described previously with a JAK2-V617F+ MPNs [10]. The first was a six-year-old girl with severe anemia and thrombocytopenia. She was found to have missense mutations in KRAS and TET2, but a normal female karyotype. She had persistent thrombocytopenia and splenomegaly and was transfusion dependent. Splenectomy was performed at 15 years of age and JAK2-V617F testing at that time revealed the mutation. She is now transfusion independent and has survived 20 years. The second patient described was a 15-year-old male with isolated thrombocytopenia. He had a frameshift mutation in RUNX1, as well as a JAK2-V617F mutation. His karyotype was more complicated, but he had no other mutations commonly found in MPNs. Fifty-one months after diagnosis, he developed mixed-lineage leukemia, received AML-based therapy, and underwent an allogeneic HSCT.

There are case reports for adult aged patients with JAK2-V617F+ MPNs that evolve into sAML [4–7]. There are no such reports of the evolution in a pediatric patient. The possibility of our patient having* de novo* AML with JAK2-V617F mutation was considered, but the multiple bone marrow examinations suggesting MPN, a subsequent examination consistent with AML, and the presence of the mutation made the diagnosis of MPN with rapid evolution into sAML more likely.
